# Cross-talk between macrophages and atrial myocytes in atrial fibrillation

**DOI:** 10.1007/s00395-016-0584-z

**Published:** 2016-09-22

**Authors:** Zewei Sun, Dongchen Zhou, Xudong Xie, Shuai Wang, Zhen Wang, Wenting Zhao, Hongfei Xu, Liangrong Zheng

**Affiliations:** 1 Department of Cardiology, The First Affiliated Hospital, College of Medicine, Zhejiang University, No. 79 Qingchun Road, Hangzhou, 310003 China; 2 Department of Cardiothoracic Surgery, The First Affiliated Hospital, College of Medicine, Zhejiang University, No.79 Qingchun Road, Hangzhou, 310003 China

**Keywords:** Atrial fibrillation, Macrophage-atrial myocyte interaction, Interleukin 1 beta, Quaking protein

## Abstract

Increased macrophage accumulation occurs in the atria of patients with atrial fibrillation (AF). However, the phenotype and functions of the macrophages in AF remain unclear. We investigated the macrophage-atrial myocyte interaction in AF patients and found that the increased macrophages were mainly pro-inflammatory macrophages (iNOS^+^, Arg1^−^). Tachypacing of HL-1 atrial myocytes also led to pro-inflammatory macrophage polarization. In addition, lipopolysaccharide (LPS)-stimulated pro-inflammatory macrophages-induced atrial electrical remodeling, evidenced by increased AF incidence and decreased atrial effective refractory period and L-type calcium currents (*I*_Ca-L_) in both canine and mouse AF models. Depletion of macrophages relieved LPS-induced atrial electrical remodeling, confirming the role of pro-inflammatory macrophages in the pathogenesis of AF. We also found that the effect of LPS-stimulated macrophages on atrial myocytes was mediated by secretion of interleukin 1 beta (IL-1β), which inhibited atrial myocyte quaking protein (QKI) expression. *IL*-*1β* knockout in macrophages restored the LPS-stimulated macrophage-induced inhibition of QKI and CACNA1C (α1C subunit of L-type calcium channel) in atrial myocytes. Meanwhile, QKI overexpression in atrial myocytes restored the LPS-stimulated macrophage-induced electrical remodeling through enhanced binding of QKI to *CACNA1C* mRNA, which upregulated the expression of CACNA1C as well as *I*_Ca-L_. In contrast, *QKI* knockout inhibited CACNA1C expression. Finally, using transcription factor activation profiling plate array and chromatin immunoprecipitation, we revealed that special AT-rich sequence binding protein 1 activated *QKI* transcription. Taken together, our study uncovered the functional interaction between macrophages and atrial myocytes in AF. AF induced pro-inflammatory macrophage polarization while pro-inflammatory macrophages exacerbated atrial electrical remodeling by secreting IL-1β, further inhibiting QKI expression in atrial myocytes, which contributed to *I*_Ca-L_ downregulation. Our study demonstrates a novel molecular mechanism underlying the pathogenesis and progression of AF and suggests that QKI is a potential therapeutic target.

**Electronic supplementary material:**

The online version of this article (doi:10.1007/s00395-016-0584-z) contains supplementary material, which is available to authorized users.

## Introduction

Atrial fibrillation (AF) is the most common cardiac arrhythmia affecting over 300 million individuals worldwide [8, 46]. Apart from compromising quality of life, AF induces stroke and heart failure, consequently increasing mortality. Currently, the major treatments for AF include rhythm control, rate control, and anti-thrombosis. However, the underlying mechanisms of AF initiation and progression are not fully understood, and, therefore, the incidence of recurrence and stroke remains high even after treatment [4].

A number of studies have uncovered a close relationship between inflammation and AF. For instance, several case–control studies showed that levels of C-reactive protein (CRP), interleukin-6 (IL-6), IL-8, and tumor necrosis factor-α (TNF-α) were significantly elevated in the atria of AF patients [3, 24, 50]. Increased infiltration of immune cells including monocytes, macrophages, and neutrophils were found in the atrial myocardium in both AF patients and in an angiotensin II-induced AF mouse model [18, 19, 22, 60]. In addition, cardiac-specific overexpression of TNF-α or tumor growth factor-β1 (TGF-β1) aggravated atrial remodeling and increased the risk of AF [7, 53, 55, 59]. In contrast, inhibition of TGF-β prevented atrial remodeling and the development of AF in a canine model [47]. Furthermore, a clinical, randomized control study found that glucocorticoids, an anti-inflammatory drug, decreased the recurrence of AF [14]. The above findings support the premise that inflammation is involved in development of AF and that anti-inflammatory therapy could be a promising AF treatment. However, there are several limitations that have restricted the clinical application of anti-inflammatory therapy for AF patients. First, the most commonly used anti-inflammatory drug, glucocorticoid, increases the risk of infection, bleeding, and hyperglycemia [26]. Second, the treatments that inhibit secretion of cytokines interfere with myocardial function [17, 33]. Therefore, it is necessary to further understand the underlying mechanisms of AF and develop more effective and specific therapeutics with little or no adverse outcomes. Recent studies suggested that macrophages are involved in the pathogenesis of AF. For instance, macrophages were increased in the atrial myocardium in patients with AF [61], and stretch of atrial myocytes, which mimics atrium enlargement, in turn also increased the number of macrophages [49]. Additionally, macrophages-induced proliferation of atrial fibroblasts in culture [6], indicating close contact of macrophages with atrial fibroblasts. Despite the progress in revealing the potential role of macrophages in AF, several questions still need to be addressed. First, which macrophage phenotype is increased in the atria? Second, do different macrophage phenotypes have different roles in AF? Third, do therapies aimed at repressing macrophage function or downstream signaling efficiently treat AF?

Quaking protein (QKI) is a RNA-binding protein that was first cloned by Ebersole et al. [15]. QKI regulates RNA splicing, export of target RNAs from the nucleus, protein translation, and maintenance of RNA stability [34]. Recent studies have found that QKI is closely associated with inflammation. For instance, Tili et al. found that QKI expression was inhibited in lipopolysaccharide (LPS)-induced activation of pro-inflammatory macrophages [58]. Fu et al. found that QKI inhibited monocyte to macrophage differentiation, an integral part of the inflammatory response [20]. Recently, bioinformatics predicted that QKI could bind to *CACNA1C* mRNA, the α1C subunit of L-type calcium channel [21], indicating a potential role for QKI in AF. However, the exact role QKI plays in the pathogenesis of AF is not known. We hypothesized that QKI regulates both macrophage function and atrial myocyte electrophysiology.

In the present study, we investigated the functional interaction between macrophages and atrial myocytes in AF. We first determined the phenotype of macrophages in the atrial myocardium in patients with sinus rhythm (SR) or AF. We then explored whether and how QKI was mediated by macrophage-atrial myocyte interaction and its involvement in the pathogenesis of AF. Our findings lend novel insight into the molecular basis underlying AF and indicate that QKI is a potential therapeutic target for treating AF in the clinic.

## Results

### AF promoted pro-inflammatory macrophage polarization

**Fig. 1:**
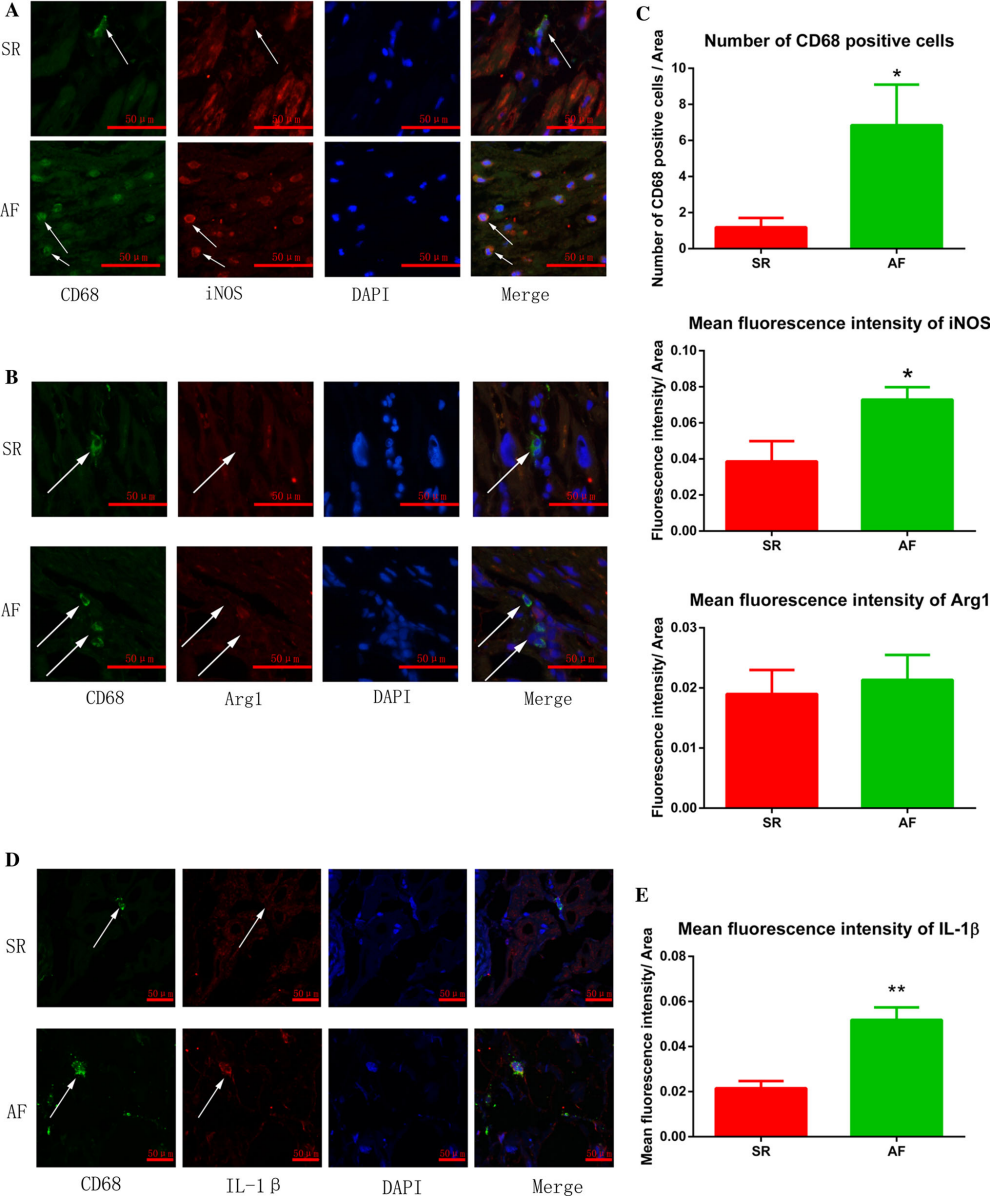
Increased atrial macrophages in AF patients were mainly pro-inflammatory. **a** Increased iNOS expression was observed in macrophages in the atria of patients with AF. **b** Arg-1 expression in macrophages was low in both SR and AF patients. **c** The statistical results of **a**, **b**. **d** Increased IL-1β expression in macrophages in the atria of patients with AF. Immunofluorescence was performed on RAA sections obtained from 11 patients with SR and 8 patients with AF. The general macrophage marker CD68, the pro-inflammatory marker iNOS, anti-inflammatory marker Arg-1, as well as IL-1β were used for staining. DAPI was used for nuclear staining. *Arrows* show macrophages. CD68, *green*; iNOS, Arg1 or IL-1β, *red*; DAPI, *blue*. *p < 0.05 SR vs. AF; **p < 0.01 SR vs. AF

**Fig. 2:**
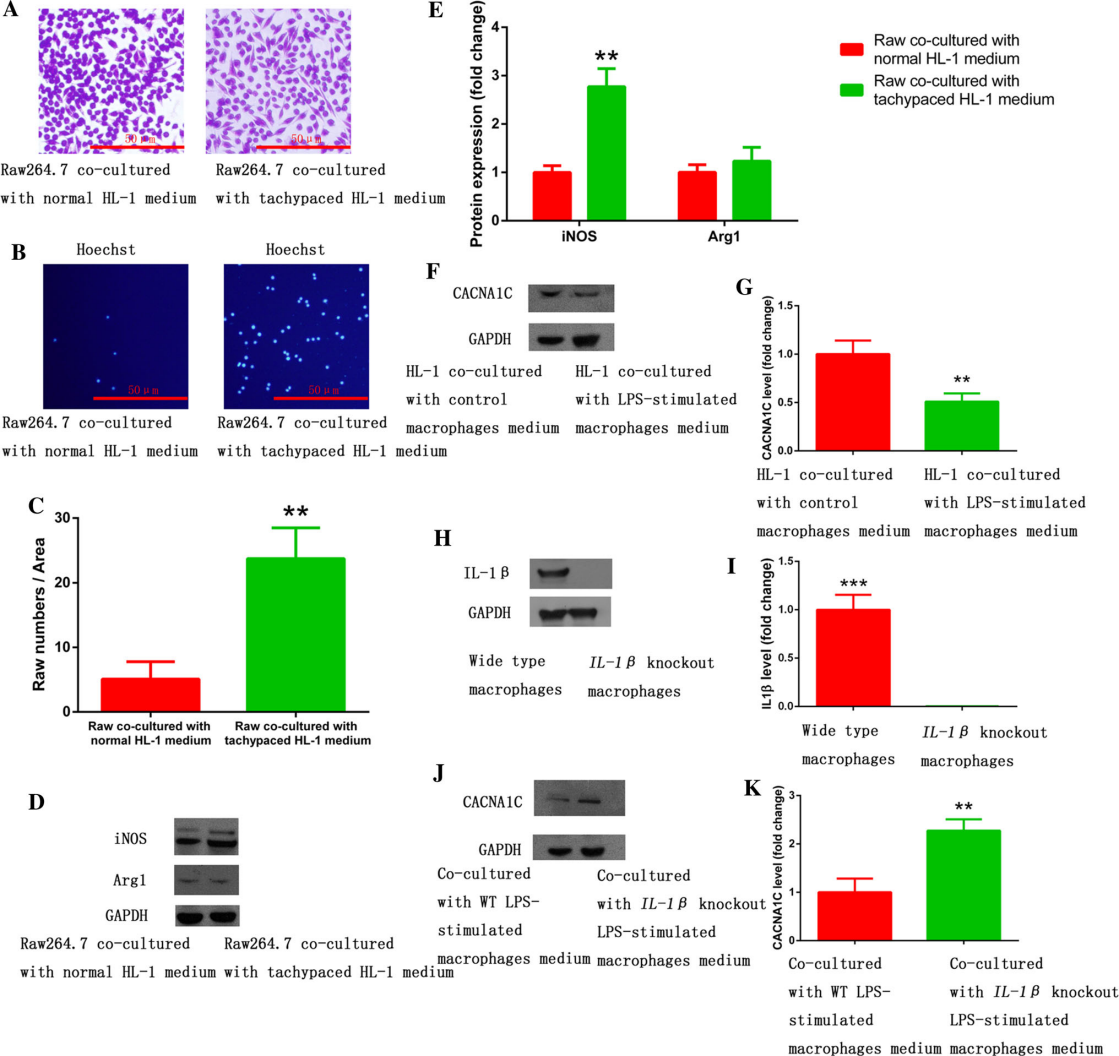
Pro-inflammatory macrophage polarization was induced by tachypaced atrial myocytes and suppressed CACNA1C expression in atrial myocytes via IL-1β secretion. **a** Raw264.7 macrophages remained round when co-cultured with normal HL-1 medium, and formed multi-synapses when co-cultured with tachypaced HL-1 medium. **b** Co-culture with tachypaced HL-1 medium increased migration of Raw264.7 cells. **c** The statistical result of **b**. **d** Co-culture with tachypaced HL-1 medium significantly elevated the expression of iNOS but not Arg1. Western blot was performed on protein lysates purified from Raw264.7 macrophages co-cultured with control medium or the medium from tachypaced HL-1 cells. GAPDH was used as a loading control. **e** The statistical result of **d**. **f** Co-culture with LPS-stimulated macrophage medium inhibited CACNA1C expression in HL-1 cells. **g** The statistical result of **f**. **h** *IL*-*1β* knockout was achieved by CRISPR/cas9 system. IL-1β expression was measured by western blot. **i** The statistical result of **h**. **j** *IL*-*1β* knockout restored CACNA1C expression repressed by LPS-stimulated macrophages. **k** The statistical result of **j**. Data were compiled from three independent experiments. **p < 0.01 vs. control group

To determine which macrophage phenotype was activated in AF, we performed immunofluorescence on RAA sections prepared from 8 AF and 11 SR patients. Using a macrophage specific marker (CD68), we found increased macrophages in RAA sections from AF patients compared to SR patients (Fig. 1a–c, white arrows). These macrophages in AF patients were iNOS-positve but Arg1-negative (Fig. 1a–c), suggesting that the cells were pro-inflammatory macrophages. These results were further confirmed by IL-1β staining, which showed increased IL-1β expression in macrophages in AF patients (Fig. 1d, e). To further confirm these findings, we used a model of atrial myocyte and macrophage co-culture. Tachypaced HL-1 atrial myocytes promoted pro-inflammatory macrophage polarization and multi-synapse formation in Raw264.7 macrophages. However, the morphology of macrophages co-cultured with control HL-1 cells remained almost round (Fig. 2a). These morphological phenotypes were further confirmed by iNOS and Arg-1 expression in Western blot. Tachypaced HL-1 cells had significantly increased iNOS but not Arg-1 expression (Fig. 2d, e). The migration assay showed that co-culture with tachypaced HL-1 cells enhanced macrophage migration (Fig. 2b, c). Taken together, these findings collectively demonstrate that AF promotes pro-inflammatory macrophage polarization.

### LPS-stimulated macrophages increased the incidence of AF through IL-1β secretion

**Fig. 3:**
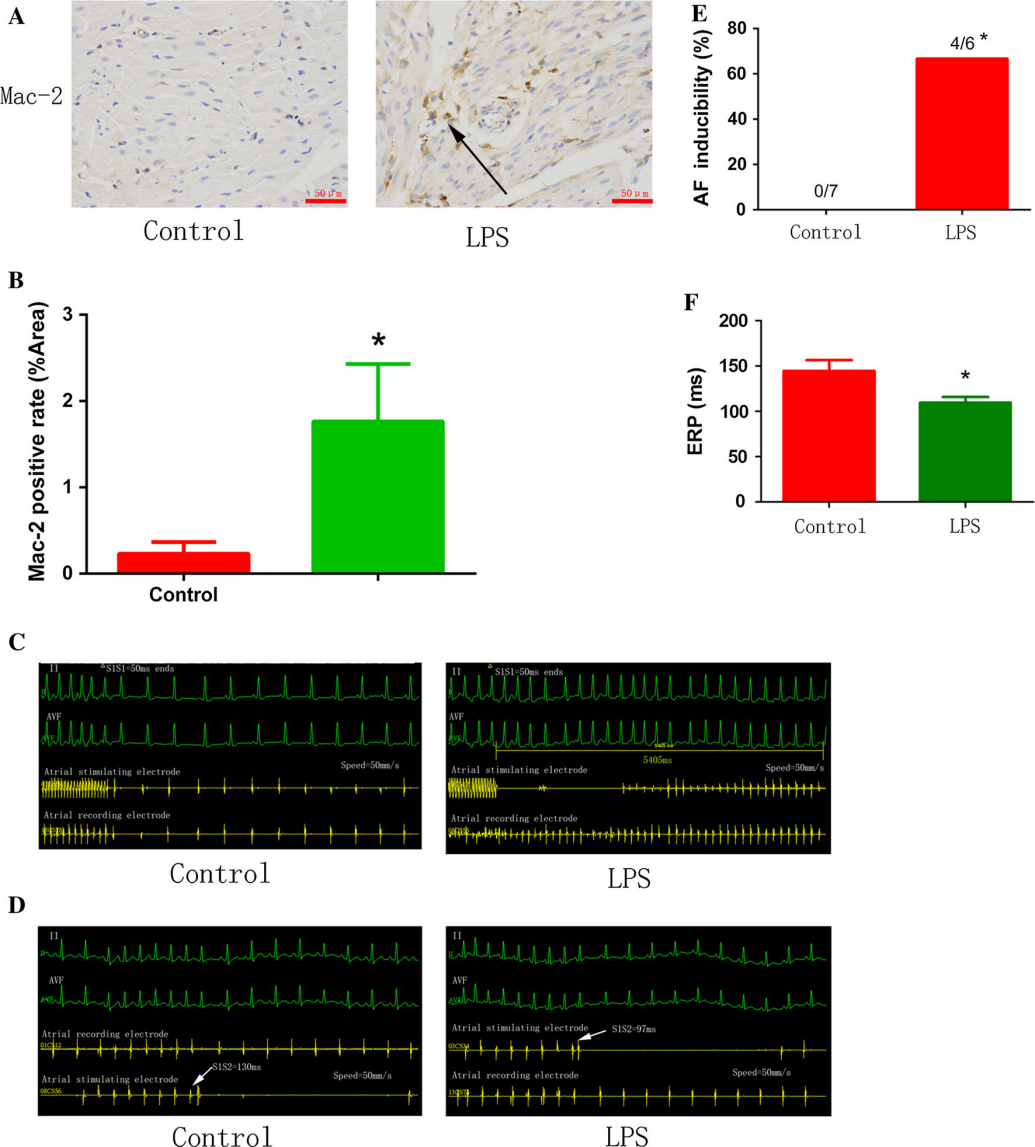
LPS-stimulated macrophages promoted atrial electrical remodeling. **a** Chronic LPS injection into canines induced pro-inflammatory macrophages. Immunostaining was performed on sections of atrial myocardium prepared from control and LPS-treated canine hearts using mac-2 antibody. *Arrow* indicates a mac-2-positive macrophage. **b** The statistical result of **a**. **c** Representative results of AF incidence in canines injected with PBS (control) or with LPS are shown. **d** Representative results of AERP in canines injected with PBS (control) or with LPS are shown. **e**, **f** Statistical analysis of AF incidence (**c**) and AERP (**d**). *n* = 7 and 6 for control and LPS-stimulated groups, respectively. *p < 0.05 vs. control group

**Fig. 4:**
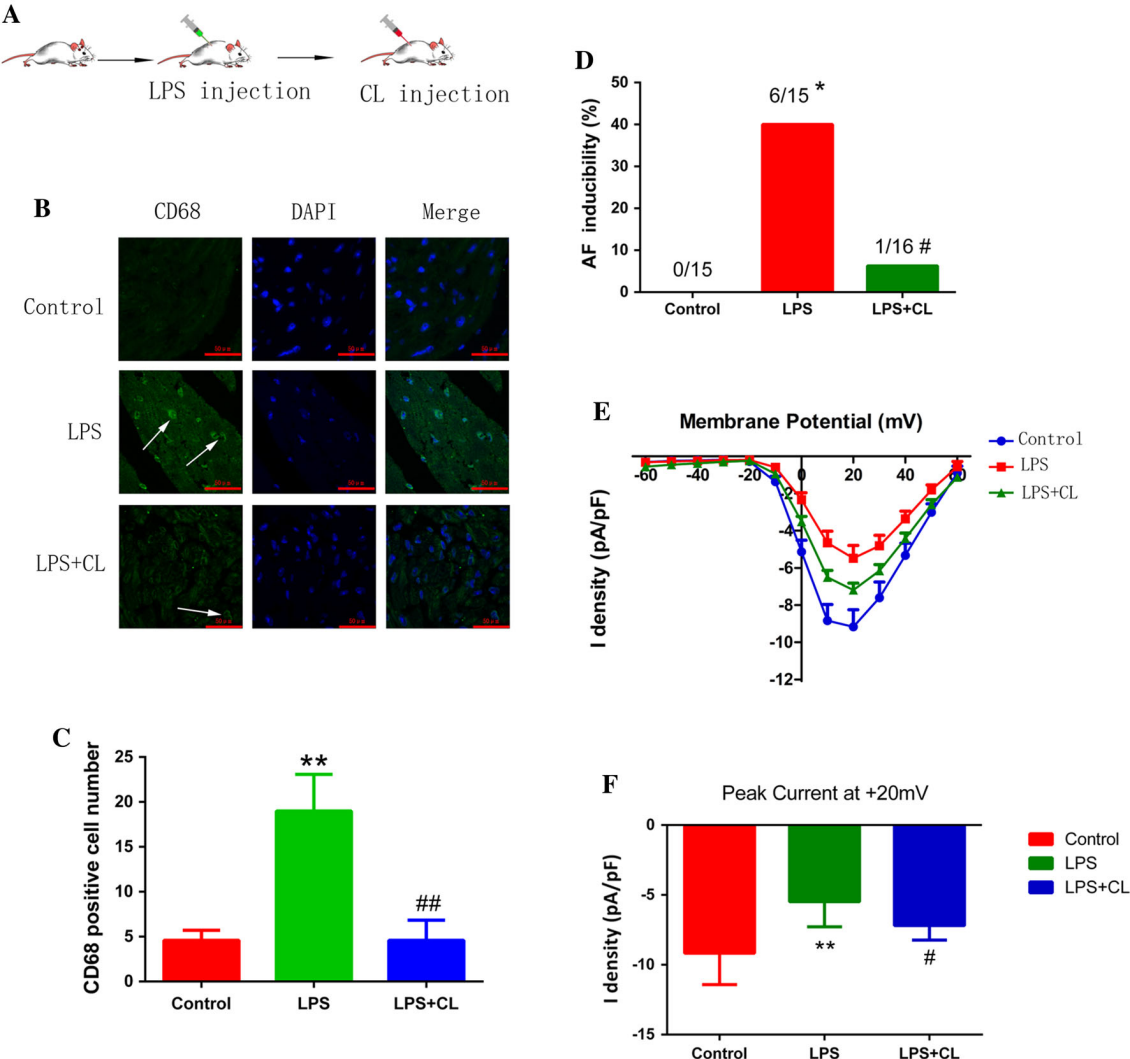
Pro-inflammatory macrophage depletion ameliorated atrial electrical remodeling in a mouse AF model. **a** Generation of chronic LPS-stimulated animal model followed by depletion of pro-inflammatory macrophages by CL. **b** LPS-induced macrophages were depleted by CL injection. (CD68, *green*; DAPI, *blue*). *Arrows* indicated CD68-positive macrophages. **c** The statistical result of **b**. **d** CL injection reduced AF incidence triggered by LPS. **e** LPS inhibited L-type calcium currents while CL partly reversed its inhibition. **f** The peak current at +20 mV of each group. *N* = 15 for control and LPS group, and *n* = 16 for LPS + CL group. *p < 0.05, **p < 0.01 vs. control group. ^#^ *p* < 0.05, ^##^ *p* < 0.01 vs. LPS group

To investigate the role of pro-inflammatory macrophages in AF pathology and its associated mechanisms, HL-1 cells were co-cultured with control medium or the medium obtained from LPS-stimulated Raw264.7 cells, which were differentiated into pro-inflammatory macrophages. Co-culture with LPS-stimulated macrophage medium inhibited CACNA1C expression in atrial myocytes (Fig. 2f, g), which was restored by *IL*-*1β* knockout in macrophages using the CRISPR/Cas9 system, suggesting that repression of CACNA1C expression by LPS-stimulated macrophage medium was mediated by IL-1β (Fig. 2h–k). We further confirmed this finding in a chronic inflammation canine model [37]. Canines received a low dose of LPS (0.1 µg/kg in 0.9 % NaCl, i.p.) once a day for 2 weeks to stimulate pro-inflammatory macrophages (Fig. 3a, b). As shown in Fig. 3c–f, LPS stimulation increased the incidence of AF and decreased atrial ERP, indicating that LPS-stimulated macrophages aggravated atrial electrical remodeling. To further confirm this finding, we generated a chronic LPS stimulation mouse model in which LPS-stimulated macrophages were depleted by CL injection (Fig. 4a). Mice were injected i.p. with LPS once a week for 8 weeks to stimulate pro-inflammatory macrophage in the atrium, and half of the LPS-treated mice were injected intravenously with CL twice a week for 2 weeks to deplete macrophages [2]. The macrophage depletion group showed a significant decrease in LPS-induced activation of pro-inflammatory macropages and electrical remodeling compared to the LPS-treated mice (Fig. 4b–d), suggesting that depletion of pro-inflammatory macrophages protected atrial myocytes from LPS-triggered electrical disorder. Mechanistically, LPS treatment downregulated the level of *I*_Ca-L_, while macrophage depletion partly restored it, as revealed by patch-clamp (Fig. 4e, f). These findings, therefore, support the notion that LPS-stimulated macrophages promote electrical remodeling of atrial cardiomyocytes in part via altering *I*_Ca-L_.

### LPS-stimulated macrophage polarization induced electrical remodeling of atrial myocytes via regulating QKI-CACNA1C signaling

**Fig. 5:**
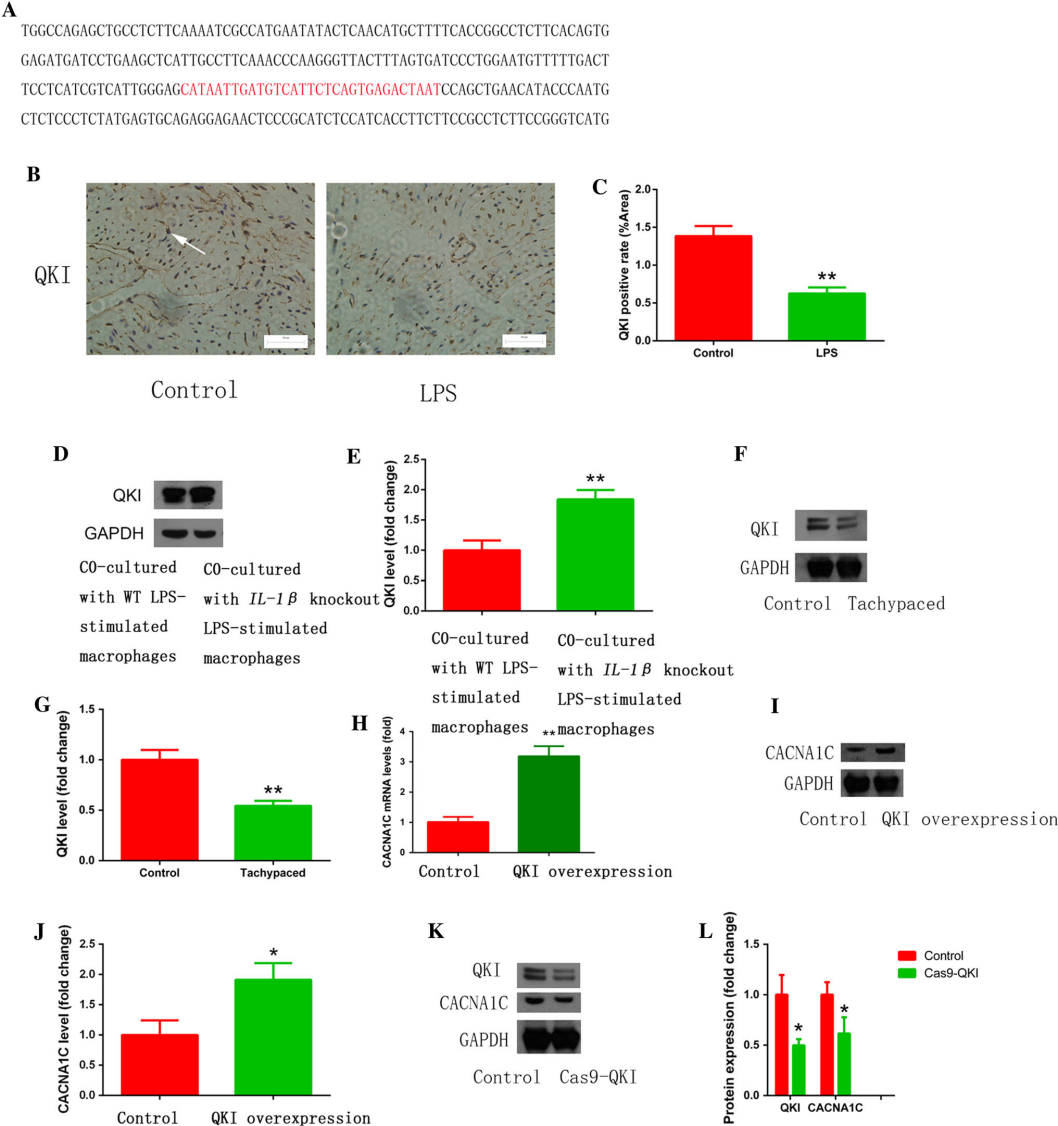
Pro-inflammatory macrophages promoted electrical remodeling through regulating QKI expression in atrial myocytes. **a** The predicted binding sequence (*red*) of QKI on *CACNA1C* mRNA. **b** LPS injection inhibited atrial QKI expression in a mouse model. Immunostaining was performed on atrial myocardium sections using a QKI antibody. *Arrow* indicates a QKI-positive atrial cardiomyocyte. **c** The statistical result of **b**. **d** Co-culture with *IL*-*1β* knockout LPS-stimulated macrophages reversed QKI expression inhibited by LPS-stimulated macrophages. Western blot was used to measure QKI expression in HL-1 cells that were co-cultured with wild type (WT) or *IL*-*1β* knockout LPS-stimulated macrophages. GAPDH was used as a loading control. **e** The statistical result of **d**. **f** HL-1 tachypacing inhibited QKI expression. Western blot was used as in **d** to measure QK1 expression in control or tachypaced HL-1 cells. **g** The statistical result of **f**. **h**, **i** QKI overexpression increased CACNA1C mRNA and protein expression. RT-qPCR (**h**) or western blot (**i**) was performed on RNA or protein lysates obtained from cultured cardiomyocytes with adenoviral-mediated expression of control or QKI. **j** The statistical result of **i**. **k** QKI knockdown decreased CACNA1C expression. **l** The statistical result of **k**. Cellular experiments were repeated three times and the sample number of mice in each group was 15. *p < 0.05, **p < 0.01 vs. control group

**Fig. 6:**
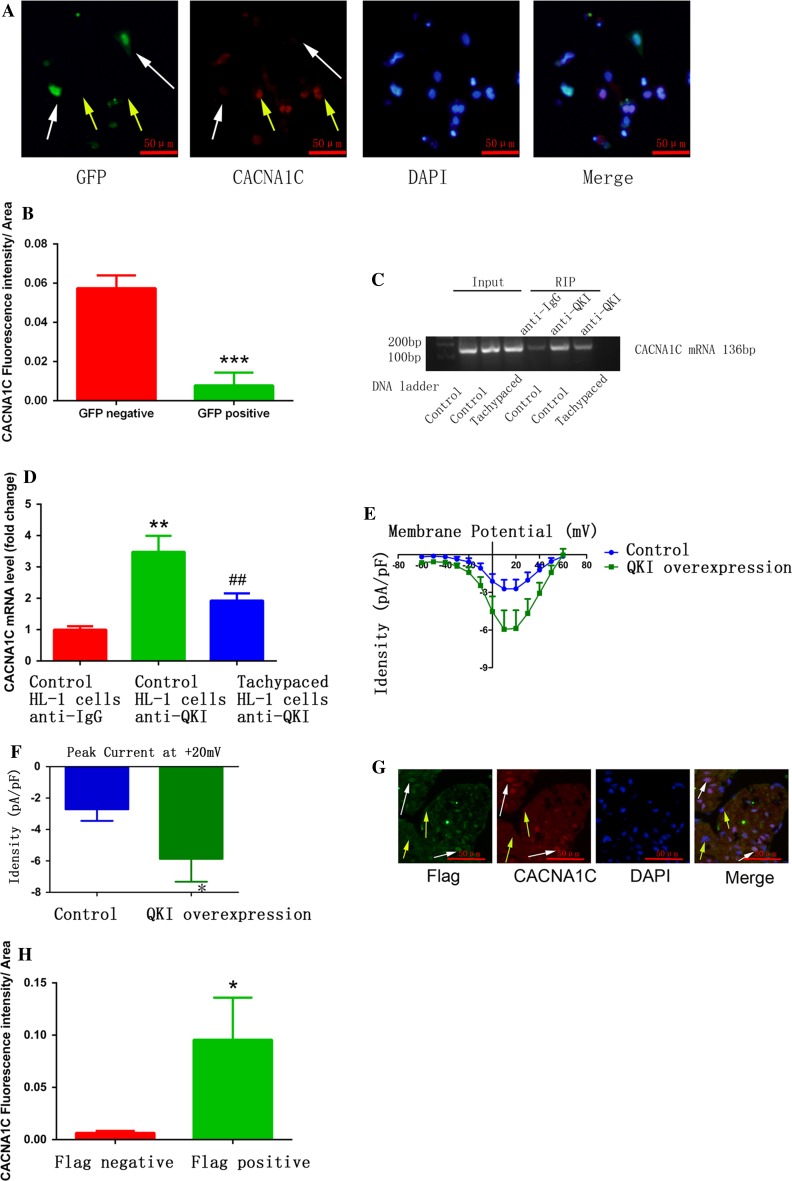
QKI mediated CACNA1C expression in atrial cardiomyocytes. **a** Primary cultured cardiomyocytes transfected with Cas9-QKI-GFP plasmid (GFP positive *white arrows*) had lower CACNA1C expression compared to GFP negative cells (*yellow arrows*). **b** The statistical result of **a**. ***p < 0.001 vs. GFP negative group. **c** QKI bound to CACNA1C mRNA. This binding was attenuated by tachypacing. RIP was performed. **d** The statistical result of **c**. **p < 0.01 control HL-1 cells anti-QKI vs. control HL-1 cells anti-IgG; ^##^ *p* < 0.01 tachypaced HL-1 cells anti-QKI vs. control HL-1 cells anti-QKI. **e** Adenoviral-mediated QKI overexpression in primary cultured cardiomyocytes increased the L-type calcium currents. **f** The peak current at 20 mV of each group. *p < 0.05 vs. control. **g** Atrial cells infected with adenovirus expressing flag-tagged QKI exhibited higher CACNA1C expression in a mouse model. **h** The statistical result of **g**. *p < 0.05 vs. Flag negative group. *White arrows* show cells transfected with plasmid or adenovirus, while *yellow arrows* show cells that were not transfected with plasmid or adenovirus. Cellular experiments were repeated three times and the sample number of mice in each group was 10

**Fig. 7:**
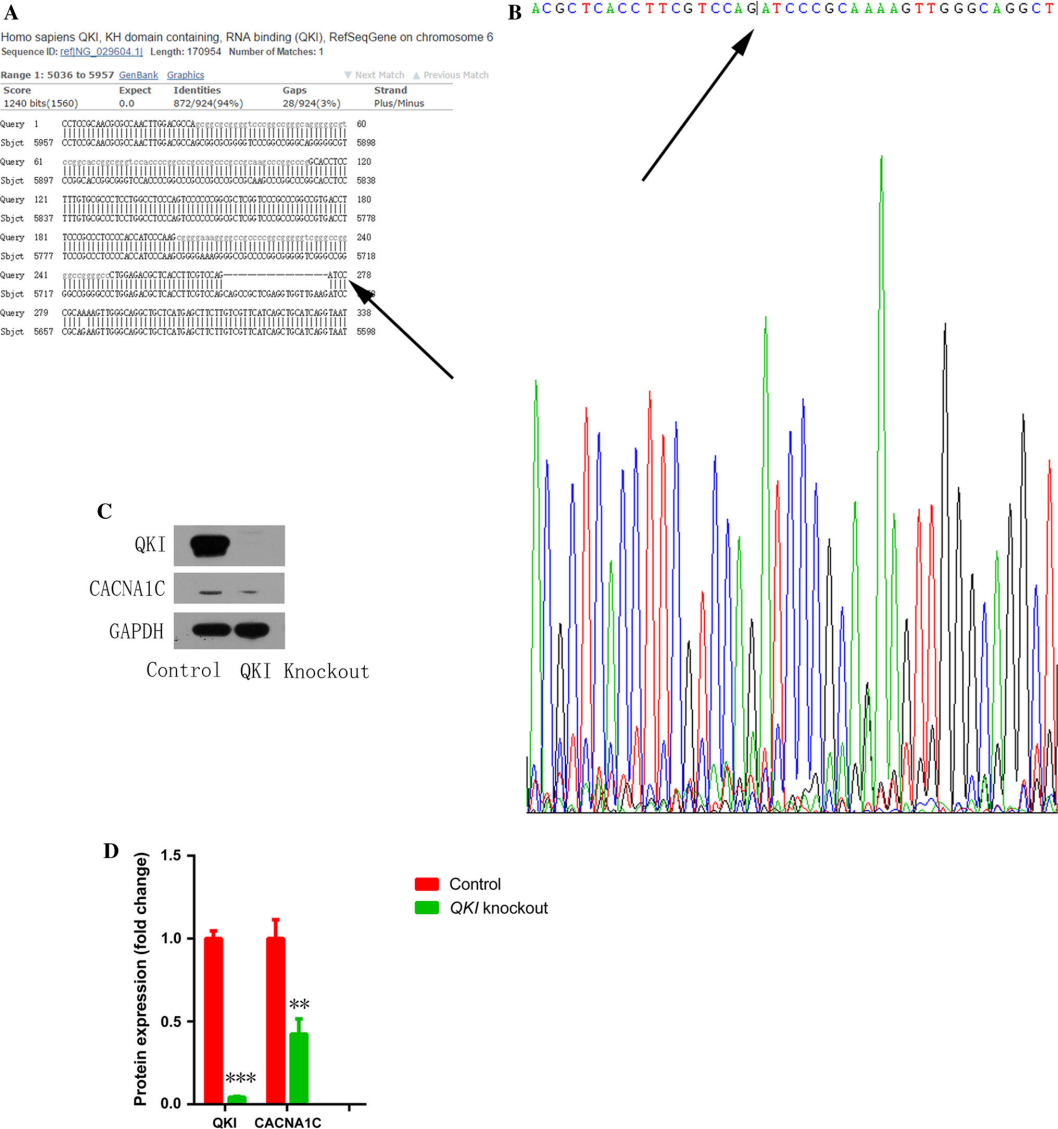
QKI mediated CACNA1C expression in HEK293T cells. **a**, **b** Transfection with Cas9-QKI led to a frameshift mutation (22 bp deletion). **c** *QKI* knockout induced the decrease of CACNA1C expression in HEK293T cells. **d** The statistical result of **c**. *Arrows* show a 22 bp deletion compared with reference sequences from NCBI databases. Experiments were repeated three times, **p < 0.01, ***p < 0.001 vs. control group

Galarneau et al. found that QKI, a RNA-binding protein that regulates RNA splicing, transportation, stability, and protein translation, bound to *CACNA1C* mRNA [21, 34] (binding sites are shown in red in Fig. 5a). In addition, Tili et al. discovered that LPS treatment downregulated QKI expression, while QKI ablation increased IL-1β expression in macrophages [58]. These findings prompted us to ask whether LPS-stimulated macrophages induce atrial myocyte electrical remodeling through regulating QKI expression. Consistent with the above findings, we found that LPS stimulation inhibited atrial QKI expression in a mouse model (Fig. 5b, c), while atrial myocytes co-cultured with *IL*-*1β* knockout macrophages increased QKI expression (Fig. 5d, e). Moreover, tachypaced HL-1 cells had decreased QKI expression (Fig. 5f, g). We also found that overexpression of QKI elevated CACNA1C mRNA and protein expression (Fig. 5h–j), while QKI knockdown mediated by the CRISPR/Cas9 system inhibited CACNA1C expression in both HL-1 (Fig. 5k, l) and primary cultured cardiomyocytes (Fig. 6a, b). RNA immunoprecipitation (RIP) assay showed that QKI bound to *CACNA1C* mRNA, which was inhibited by tachypacing (Fig. 6c, d). To further confirm the effect of QKI on CACNA1C expression, we overexpressed QKI in a mouse model via adenovirus transduction and found that infected atrial myocytes (white arrows) had higher CACNA1C expression compared to non-infected cells (yellow arrows) (Fig. 6g, h). We also investigated the effect of QKI on *I*_Ca-L_, and found that QKI overexpression increased *I*_Ca-L_ (Fig. 6e, f). We further confirmed the relationship between QKI and CACNA1C by *QKI* knockout in HEK293T cells. Compared with the reference sequence from NCBI databases, transfection with Cas9-QKI led to a frameshift mutation (22 bp deletion, Fig. 7a, b) which further induced the decrease of CACNA1C expression (Fig. 7c, d). Taken together, we conclude that QKI-CACNA1C signaling is involved in mediating LPS-stimulated macrophage-induced electrical remodeling of atrial cardiomyocytes.

### Satb1 mediated QKI expression

**Fig. 8:**
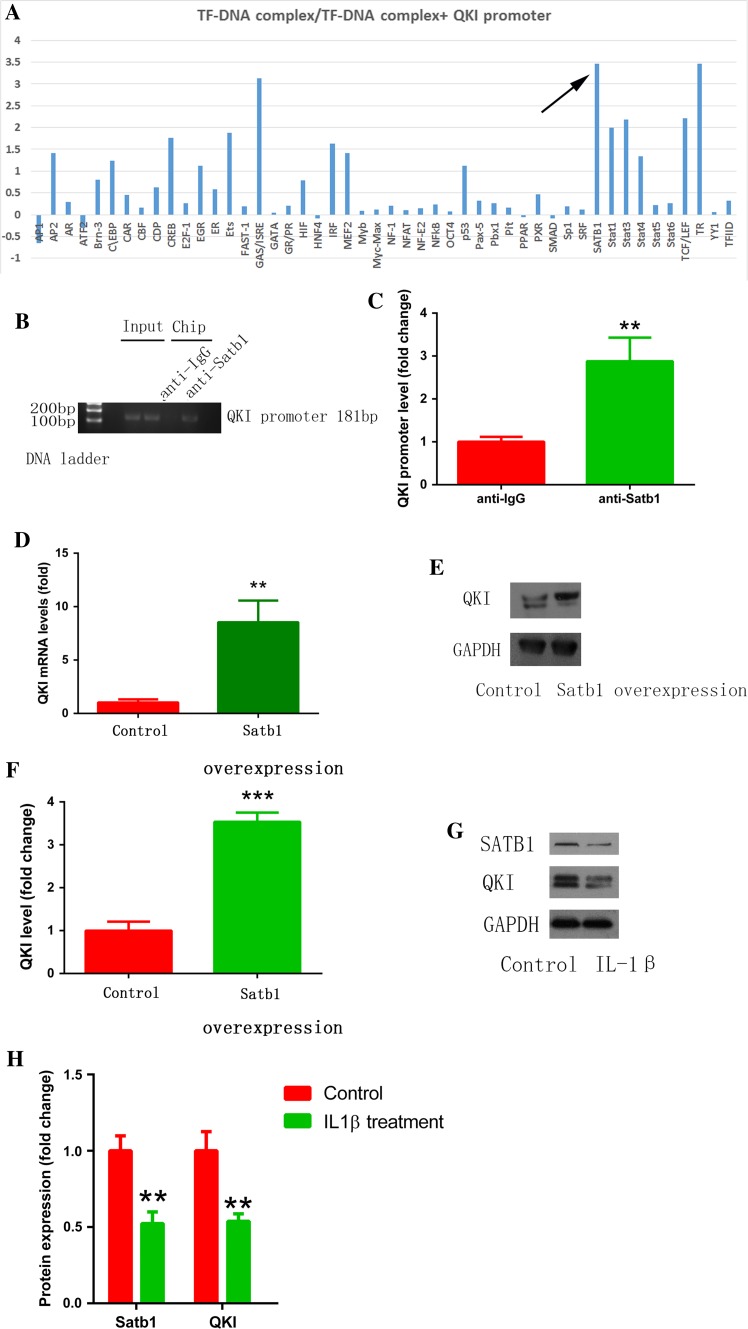
Satb1 mediated QKI expression. **a** Transcription factor binding screening assay showed the binding of Satb1 to the QKI promoter (*arrow*). **b** ChIP assay confirmed the binding of Satb1 to the QKI promoter. **c** The statistical result of **b**. **d**, **e** Satb1 overexpression increased QKI mRNA and protein expression. **f** The statistical result of **e**. **g** IL-1β stimulation (30 pg/ml, 48 h) decreased Satb1 and QKI expression. **h** The statistical result of **g**. **p < 0.01, ***p < 0.001 vs. control. Experiments were repeated three times

Next, we investigated the upstream regulation of QKI expression by performing transcription factor screening. As shown in Fig. 8a, relative light units (RLU) were significantly decreased in the Satb1 probe + nuclear protein + QKI promoter group compared to the Satb1 probe + nuclear protein group. This suggests that Satb1 binds to the QK1 promoter. Indeed, ChIP assay showed that anti-Satb1antibody but not IgG precipitated a significant amount of the targeted chromatin fragment of the QK1 promoter (Fig. 8b, c). Functionally, overexpression of Satb1 increased QKI mRNA and protein expression in cultured cardiomyocytes (Fig. 8d–f). We further confirmed the role of Satb1 in macrophage–myocyte interaction by IL-1β stimulation (30 pg/ml for 48 h) and found that IL-1β decreased Satb1 and QKI expression (Fig. 8g, h). Taken together, we argue that QK1 expression is mediated, in part, by Satb1. Moreover, we measured the expression of Satb1 and QKI in human samples and found that both were decreased in patients with AF (Supplementary Fig. 2).

### TNF-α was involved in electrical remodeling induced by LPS-stimulated macrophages

Apart from IL-1β, LPS-stimulated macrophages can secrete several other cytokines including TNF-α [45]. We then investigated whether TNF-α was involved in electrical remodeling. As shown in Supplementary Fig. 1A, LPS stimulation induced TNF-α secretion. Since TNF-α can inhibit connexin 40 expression [39, 55], TNF-α may be another candidate that is involved in macrophage-induced electrical remodeling given that connexin 40 expression was downregulated in AF and connexin 40 transfer using adenovirus inhibited AF inducibility [29]. Consistent with the above observations, LPS stimulation inhibited connexin 40 expression while TNFα antibody partly abolished this inhibitory effect, indicating the involvement of TNF-α in electrical remodeling induced by LPS-stimulated macrophages (Supplementary Fig. 1B, C).

## Discussion

**Fig. 9:**
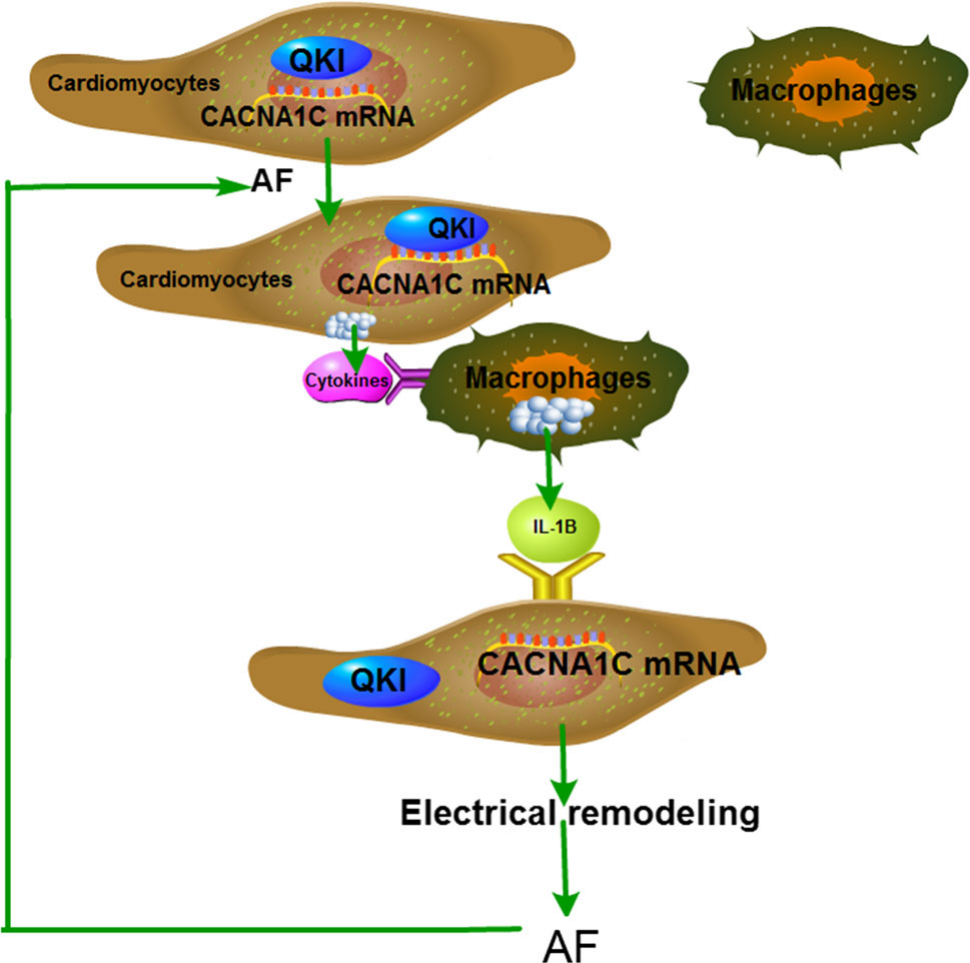
Working model of cellular signaling underlying the functional interaction between atrial cells and macrophages in AF. AF promotes pro-inflammatory macrophage polarization by secreting cytokines. Pro-inflammatory macrophages induce atrial electrical remodeling by secreting IL-1β, which subsequently inhibits QKI expression in atrial myocytes and decreases QKI-CACNA1C mRNA binding, consequently suppressing L-type calcium currents, which promotes atrial electrical remodeling and exacerbates AF

The main findings of the present study are: (1) increased pro-inflammatory macrophages were found in the atria of AF patients, and HL-1 cell tachypacing induced pro-inflammatory macrophage polarization; (2) pro-inflammatory macrophage polarization played a major role in atrial electrical remodeling as evidenced by the increased incidence of AF in chronic LPS treatment in mice and canines, which was inhibited by the depletion of LPS-stimulated macrophages; (3) the effect of LPS-stimulated macrophages on electrical remodeling was mediated by IL-1β secretion, which inhibited QKI expression in atrial myocytes; (4) QKI bound to *CACNA1C* mRNA and regulated the level of *I*_Ca-L_; and (5) the transcription factor Satb1 mediated QKI expression. Our findings are summarized in the working model shown in Fig. 9.

Although previous studies have reported increased macrophage accumulation in AF, the macrophage phenotype and functions were unknown. Our study revealed that the increased macrophages in AF patients were mainly pro-inflammatory macrophages. This phenomenon can be explained by a number of possibilities. First, since the macrophages are pro-inflammatory [23], increased inflammation in AF may be attributed, at least in part, to increased pro-inflammatory macrophages. Second, several systemic diseases, including obesity and hypertension, are associated with low-grade inflammation and pro-inflammatory macrophage polarization. Mice fed high-fat diets also showed an increase in pro-inflammatory macrophages in adipose tissue [41]. Actually, a clinical investigation showed that inflammatory activity in epicardial adipose tissue was associated with AF [43]. Hypertension, another major cause for AF, also led to increased pro-inflammatory macrophages, and the anti-hypertensive, hemin, achieved its function via enhancing anti-inflammatory and suppressing pro-inflammatory macrophages [48]. Third, the levels of pro-inflammatory cytokines secreted by pro-inflammatory macrophages, including TNF-α, IL-1β, and IL-6 [45], were elevated in patients with AF [24, 38, 50]. In contrast, the anti-AF factor, HSP27, played a protective role via enhancing IL-10, one of the anti-inflammatory cytokines secreted by anti-inflammatory macrophages [28].

In the present study, we found that LPS-stimulated macrophages led to atrial electrical remodeling. Mechanistically, LPS-stimulated macrophages secrete cytokines including TNF-α, IL-1β, and IL-6, all of which can promote AF. For example, TNF-α induced abnormal Ca^2+^ handling and arrhythmogenicity in pulmonary vein cardiomyocytes [35], and perfusion with IL-6 resulted in the appearance of AF [44]. Although the relationship between IL-1β and AF was unclear, our findings support the premise that both IL-1β and TNF-α are involved in mediating pro-inflammatory macrophage-induced electrical remodeling.

Previously, QKI was shown to be downregulated in macrophages in response to LPS stimulation [58], and QKI overexpression elevated IL-10 expression, which is one of the cytokines secreted by anti-inflammatory macrophages. These observations indicated that QKI mediates the balance of pro-inflammatory/anti-inflammatory macrophages [58], altering the inflammation state. Consistent with the above findings, our observation suggested that LPS-stimulated macrophage-induced electrical remodeling was associated with reduced QKI expression. Bioinformatics predicted that QKI could bind to *CACNA1C* mRNA, which was confirmed by RIP in our study. Furthermore, we showed that QKI not only upregulated CACNA1C expression, but also enhanced the level of *I*_Ca-L_, which plays important roles in the pathogenesis of AF. For example, Barana et al. reported that microRNA-21 participated in AF by inhibiting *I*_Ca-L_ and CACNA1C expression [1], and Lu et al. also showed that microRNA-328 contributed to the adverse atrial electric remodeling in AF through targeting *CACNA1C* and *CACNB1* [40]. Therefore, QKI inhibited electrical remodeling via several potential mechanisms: regulating the balance of pro-inflammatory/anti-inflammatory macrophages, increasing CACNA1C expression, and potentiating *I*_Ca-L_ activity. Finally, we found that the transcription factor Satb1 bound to the QKI promoter and mediated QKI expression. IL-1β stimulation decreased Satb1 and QKI expression. This result was consistent with a previous finding, which showed that LPS suppressed Satb1 expression through miRNA-155, a pro-inflammatory microRNA [5]. Given that QKI was also the target of miRNA-155 [58], we hypothesized that there was a potentially functional link between Satb1 and QKI, which requires further investigation.

Our study found that inflammation was involved in AF; however, it should be noted that positive results with anti-inflammation drugs [12, 13] were accompanied by many negative studies [10, 32]. This can be explained as follows: (a) in some studies anti-inflammatory treatment did not result in the inhibition of inflammation. For example, Darghosian et al. found that omega-three polyunsaturated fatty acids had no effect on AF [10]. However, in their work the omega-three polyunsaturated fatty acids did not cause a meaningful downregulation of inflammation marker levels. Thus, this result cannot explain the association of AF and inflammation. (b) As discussed by Hu et al. [27], AF recurrence was influenced by the individual center apart from the disease per se. Thus, a potential bias might occur when studying inflammation and AF, because increased inflammation may arise from extensive ablation. Furthermore, the potential importance of inflammation in AF is evidenced by an ongoing clinical trial with the IL-1β inhibitor, Canakinumab, and AF [52].

Our study has several limitations. First, we observed increased accumulation of macrophages in the atria in AF; however, the source of macrophages remains unclear. Future investigation will determine if the macrophages originated from recruited circulating monocytes, onsite proliferation of macrophages, or differentiation of resident macrophage progenitors [9, 36]. Second, we mainly studied pro-inflammatory macrophage-induced electrical remodeling. Apart from electrical remodeling, structure remodeling is another important characteristic of AF [62]. Previous studies have shown that TNF was associated with structural remodeling [27], indicating that pro-inflammatory macrophages also play a role in AF structural remodeling, which warrants further exploration. Third, we used CL by intravenous injection and found that depletion of macrophages prevented AF; however, whether this effect was achieved by depletion of macrophages in atria or other tissues remains unclear. Thus, depletion of local macrophages could be more important and requires further clarification [42]. Fourth, while pro-inflammatory macrophages promote AF, the function of anti-inflammatory macrophages in AF remains unclear. Fifth, other reports showed that strategies to accelerate transition from pro-inflammatory to anti-inflammatory macrophages have successfully promoted angiogenesis, reduced infarct size, and prevented left ventricular remodeling [11, 25]. As described above, QKI was downregulated in pro-inflammatory macrophages while QKI overexpression increased IL-10 secretion [58], indicating that QKI may serve as a switch molecule. Thus, understanding the macrophage switch from pro-inflammatory to anti-inflammatory in AF and whether QKI plays a role in this phenotypic switch merits further investigation. Furthermore, recent studies indicated that inhibition of CD40 induced macrophages polarization from pro-inflammatory towards anti-inflammatory. Hence, CD40 could be another target to explore the effects of different macrophage phenotypes on AF [30]. Finally, since the main functions of QKI are regulating RNA splicing, transportation, stability, and protein translation, the exact mechanisms involving QKI-mediated CACNA1C expression need to be further investigated.

In conclusion, our study demonstrates that AF promotes pro-inflammatory macrophage polarization, and that pro-inflammatory macrophages further induce atrial electrical remodeling through secreting IL-1β. Increased release of IL-1β suppresses QKI expression in atrial myocytes, leading to decreased L-type calcium currents. Our study uncovers a novel molecular mechanism for AF and points to QKI as a potential therapeutic target.

## Materials and methods

### Reagents

LPS, Claycomb Medium, fetal bovine serum (FBS), norepinephrine, and l-glutamine were purchased from Sigma-Aldrich (USA). Dulbecco’s modified Eagle medium (DMEM) was from Thermo Fisher Scientific (USA). QKI encoding vector and adenoviruses were purchased from Vigene Biosciences (China). JetPRIME and JetPEI-Macrophage transfection reagents were from Polyplus Transfection (France). Antibodies against CD68, inducible nitric oxide (iNOS), arginase-1 (Arg-1), QKI, and IL-1β were obtained from Abcam (USA). Antibody against CACNA1C was purchased from Santa Cruz (USA). RNA immunoprecipitation kit was from Millipore (USA). Chromatin immunoprecipitation (ChIP) kit was obtained from CST (USA). Clodronate Liposomes (CL) was from http://clodronateliposomes.com (The Netherlands). Promoter-Binding TF Profiling Plate Array kit was purchased from Signosis (USA).

### Human samples

Right atrial appendages (RAA) were obtained as surgical specimens from 11 patients with SR and 8 patients with persistent AF undergoing cardiac valve replacement. The detailed clinical characteristics of these patients are described in Table 1. No patients involved in this study had a history of myocardial infarction, febrile disorders, systemic inflammatory diseases, malignancy or chronic renal failure.

**Table 1:** Demographic and basic clinical characteristics of patients involved in the study

	Sex	Age	AF history (months)	HD	EF (%)	LAD (mm)	Drug therapy
SR							
	M	66		CHD, MR	61	30	B, C, D, N, W
	M	60		AR	70	47	D, W
	M	68		MR	61	36	A, C, D, N, W
	M	56		MR	53	37	A, C, D, N, W
	M	63		AR, MS	59	38	D, N, W
	F	38		MR, MS, AR	59	35	B, D, W
	F	72		AR, AS	52	28	B, C, D, N, W
	F	61		MR, MS	55	32	B, C, D, N, W
	F	45		MS	69	35	D, W
	F	33		MR	61	25	D, W
	F	45		ASD	73	32	D, N
Total	5M/6F	55.18 ± 3.91			61.18 ± 2.08	34.09 ± 1.76	
AF							
	M	59	24	AR	60	31	C, D, N, W
	M	60	84	MR, MS, AR, AS	61	46	C, D, N, W
	F	36	67	MR, MS	61	65	D, W
	F	67	12	MR	50	41	D, N, W
	F	62	96	MR, AR, AS	66	33	B, C, D, N, W, AD
	F	43	6	MR, MS, AR	70	40	B, D, W, AD
	F	64	12	MR, MS	69	54	C, D, N, W
	F	54	1	MR, MS	62	41	D, W
Total	2M/6F	55.63 ± 3.82	37.75 ± 13.54		62.38 ± 2.22	43.88 ± 3.93*	

*AF* atrial fibrillation, *HD* heart disease, *EF* ejection fraction, *LAD* left atrial dimension, *SR* sinus rhythm, *CHD* coronary heart disease, *MR* mitral regurgitation, *MS* mitral stenosis, *AR* aortic regurgitation, *AS* aortic stenosis, *ASD* atrial septal defect, *A* ACE or ARB inhibitors, *B* β-blockers, *C* calcium-channel blockers, *L* loop diuretics, *N* nitrates, *W* warfarin, *AD* antiarrhythmic drug

* *p* < 0.05 vs. SR

### Experimental animals

C57BL/6 mice were obtained from the Shanghai Laboratory Animal Center of the Chinese Academy of Sciences (China). All mice were 8 weeks old with an average weight of 25–30 g. Beagle dogs were obtained from Huishan Laboratory Animal Center (China) with an average weight of 8–10 kg. All mice and dogs were housed in the animal facility, which was maintained at 20–25 °C, 55 % relative humidity, with an automatic 12 h light/dark cycle. All animals received a standard laboratory diet and tap water ad libitum, and were acclimated for 1 week before experimentation.

### Generation of animal model with chronic inflammation

The chronic inflammation animal model was generated by recurrent exposure to subclinical LPS as described [37]. Briefly, mice were injected intraperitoneally (i.p.) with saline (control) or LPS (10 mg/kg) once a week for 2 months. To deplete macrophages, CL was injected through the tail vein at a dose of 10 μl/g twice a week for 2 weeks. Dogs received a low dose of LPS (0.1 µg/kg in 0.9 % NaCl, i.p.) once a day for 2 weeks.

### In vivo electrophysiology and programmed stimulation

In mice, in vivo electrophysiology and programmed stimulation experiments were conducted as previously described [16]. Briefly, mice were anesthetized under isoflurane anesthesia (1.5 % vol/vol) and a subcutaneous administration of 0.03 mg/kg buprenorphine hydrochloride. The following signs were monitored to establish the adequacy of anesthesia: (1) no limb and palpebral withdrawal reflexes; (2) stable respiration and heart rates. Then the subdermal needle electrodes were placed in all four legs to make a lead II conformation. A 1.1F electrophysiology catheter containing eight electrodes (Scisense Inc., Canada) was inserted through the jugular vein. The correct position was confirmed by obtaining a sole ventricular signal in the distal lead and a predominant atrial signal in the proximal lead. AF inducibility was determined using burst pacing in the right atrium. In detail, three trains of 2 s burst pacing were given as follows: the first 2 s burst was applied at a cycle length of 40 ms with a pulse duration of 5 ms. After 3 min of stabilization, the second 2 s burst was set at a cycle length of 20 ms with a pulse duration of 5 ms. After another 3 min of stabilization, the final 2 s burst was given at a cycle length of 20 ms with a pulse duration of 10 ms. AF was defined as a rapid and irregular atrial rhythm with irregular R–R intervals for at least 1 s on the surface electrocardiogram (ECG). All ECG data were acquired using a cardiac electrophysiology stimulator and multichannel electrophysiological recording system (Scisense Inc., Canada). After measurement, the mice were immediately sacrificed by cervical dislocation.

Canines were anesthetized with sodium pentobarbital (initial bolus 30 mg/kg i.v., 50–100 mg as needed for maintenance). The following signs were monitored to establish the adequacy of anesthesia: (1) no limb and palpebral withdrawal reflexes; (2) stable respiration and heart rates. Atrial effective refractory period (AERP) was determined by programmed stimulation at RAA, which consisted of eight consecutive stimuli (S1S1 = 250 ms) followed by a premature stimulus (S1S2). The S1S2 intervals were decreased from 200 ms to refractoriness initially by a 2 ms decrement. The atrial ERP was defined as the longest S1–S2 interval that failed to induce atrial depolarization. AF inducibility was also measured by burst pacing. Two trains of 120 s burst pacing were given as follows: first, a 120 s burst was applied at a cycle length of 100 ms. After 5 min of stabilization, the second 120 s burst pacing was given at a cycle length of 50 ms. AF was defined as irregular atrial rates faster than 500 beats/min associated with irregular atrio-ventricular conduction lasting longer than 5 s. After measurement, canines were euthanized by removal of the heart.

### Cell culture

Murine atrial myocytes, HL-1, were provided by Dr. Chen and Dr. Wang’s lab (Nanjing medical university, China) with the permission of Dr. Claycomb (LSU Health Sciences Center, USA). The cells were cultured with Claycomb Medium supplemented with 10 % FBS, 100 U/ml penicillin/streptomycin, 0.1 mM norepinephrine, and 2 mM l-glutamine. HL-1 cell tachypacing was performed using a cell pacing system (Ionoptix, USA). HL-1 cells were subjected to rapid stimulation for 24 h at 5 Hz (18 V, 4 ms) [56].

Raw264.7 macrophages and HEK293T cells were purchased from American Type Culture Collection (ATCC, USA) and cultured with DMEM supplemented with 10 % FBS. To develop pro-inflammatory macrophages, 100 ng/ml LPS was added for 16 h as previously described [54].

Primary neonatal rat cardiomyocytes were isolated from 1-day-old rats as described [57]. Briefly, hearts were minced and digested with trypsin and collagenase, and the isolated cells were pre-plated twice for 60 min to eliminate fibroblasts. The non-adherent myocytes were then plated in plating medium containing 199 medium supplemented with HEPES, MEM non-essential amino acids, glucose, glutamine, 10 % FBS, vitamin B12, penicillin, and streptomycin on fibronectin-coated plates.

Macrophages and atrial myocytes were co-cultured in exchanging medium. In brief, RAW264.7 cells were treated with 100 ng/ml LPS for 16 h, then washed with PBS, and cultured in fresh medium for another 24 h. Thereafter, the medium was collected and used for culturing HL-1 cells. Similarly, HL-1 cells were tachypaced for 24 h. Thereafter, the medium was collected and used for culturing RAW264.7 cells.

### Transient transfection and in vivo gene transfer

DNA transfection was performed using jetPRIME or jetPEI-Macrophage for cardiomyocytes or macrophages, respectively. For cardiomyocytes, 2 μg DNA was added into 200 μl jetPRIME buffer, followed by addition of 4 μl jetPRIME. After a 10 min incubation, the transfection mix was added into each well. For macrophages, 6 μg DNA was added into 50 μl of 150 mM NaCl, followed by addition of 50 μl jetPEI-Macrophage solution. After a 30 min incubation, the transfection mix was added into each well.

In vivo gene transfer was conducted by injecting adenoviruses at a dose of 10^9^ plaque forming units (pfu) in 50 μl PBS through the tail vein [31].

### Genome editing with CRISPR/Cas9 system

Genome engineering was performed using CRISPR/Cas9 systems according to published protocols [51]. Briefly, guide RNA (sgRNA) was designed and inserted into pSpCas9 (BB)-2AGFP or pSpCas9 (BB)-2A-Puro vector. The sgRNA sequences targeting QKI were: sgRNA top, CACCGGATCTTCAACCACCTCGAG; sgRNA bottom, AAACCTCGAGGTGGTTGAAGATCC. The sgRNA sequences targeting *IL*-*1β* were: sgRNA top, CACCGAGCACCTAAGTCCCTAGGTT; sgRNA bottom, AAACAACCTAGGGACTTAGGTGCTC. The plasmids were amplified in *E. coli* strain, purified, sequenced, and used for transfection into macrophages, HEK293T cells or cardiomyocytes as described above. For macrophages and HEK293T cells, cells transfected with Cas9-IL1β-puro or Cas9-QKI-puro vector were further treated with puromycin (10 µg/ml) for 72 h. Surviving cells were used for monoclonal formation. *QKI* and *IL*-*1β* knockout were determined by sequencing and Western blot. Since single HL-1 cell was not able to form monoclones, we used a mixture pool for QKI knockdown. In detail, HL-1 cells transfected with Cas9-QKI-puro vector were treated with puromycin and surviving cells were used for analysis as a mixture pool. QKI knockdown was measured by Western blot. Since primary cultured cardiomyocytes have no proliferative capability, cells were transfected with Cas9-QKI-GFP vector, and the GFP-positive and GFP-negative cells were used for analysis.

### Immunohistochemistry and immunofluorescence

Immunohistochemistry and immunofluorescence were performed as described [57, 63]. The primary antibodies used were: CD68 (1:100, Abcam), iNOS (1:50, Abcam), Arg-1 (1:100, Abcam), QKI (1:200, Abcam), and CACNA1C (1:50, Santa Cruz).

### RNA immunoprecipitation

RNA immunoprecipitation was performed according to the manufacturer’s protocol. Briefly, 2 × 10^7^ control or tachypaced HL-1 cells were collected and lysed, and the lysates were incubated with magnetic bead-QKI or IgG antibody with rotation at 4 °C overnight. Samples were then digested by proteinase K and RNA was purified using phenol–chloroform–isoamyl alcohol. RT-PCR was performed on purified RNA. The primers used for identifying QKI binding sequence were as follows: forward-5′GCTCATTGCCTTCAAACC3′; reverse-5′GATGGAGATGCGGGAGTT3′.

### Real-time PCR

Real-time PCR was conducted as described previously [57]. The primers used were as follows: *QKI*: forward-5′AGGCAAAGGCTCAATGAGGG3′, reverse-5′CCTGGGCAGTTGGTGATTT3′; *CACNA1C*: forward-5′TCCCGAGCACATCCCTACTC3′, reverse-5′ACTGACGGTAGAGATGGTTGC3′; *β*-*actin*: forward-5′GGCTGTATTCCCCTCCATCG3′, reverse-5′CCAGTTGGTAACAATGCCATGT3′.

### ChIP

ChIP was performed according to the manufacturer’s instructions. Briefly, HL-1 cells were fixed with 1 % formaldehyde for 10 min at 37 °C. Chromatin was digested with 5 µl nuclease at 37 °C for 20 min, yielding DNA fragments of 200–800 bps. After preclearing with protein A/G agarose at 4 °C for 1 h, samples were incubated with 10 μg of Satb1 antibody or IgG (as a control) with rotation at 4 °C overnight. The reverse crosslink was reversed by incubation of the sample in a 5 M NaCl and proteinase K solution at 65 °C for 2 h. The precipitated DNA was purified using spin columns, and the purified DNA was subjected to PCR analysis. The primers used for PCR to identify the Satb1 binding sequence were as follows: forward-5′CCCAGTGAAGCAACAGGA3′; reverse-5′GGAATCCCGGCGGGACTC3′.

### Whole-cell patch-clamp

Whole-cell patch-clamp was performed using a HEKA EPC10 amplifier (HEKA, Germany). The bath solution contained 140 mM TEA-Cl, 2 mM MgCl2, 10 mM CaCl_2_, 10 mM HEPES, and 5 mM glucose at pH 7.4 (TEA-OH). The internal solution contained 120 mM CsCl 120, 1 mM MgCl_2_,10 mM HEPES, 4 mM Mg-ATP,10 mM EGTA, and 0.3 mM Na2-GTP at pH 7.2 (CsOH). The pipettes were created from capillary tubing (Sutter Instruments, USA) and had resistances of 4–6 MΩ under these solution conditions. All recordings were carried out at room temperature. Cav IV-curve was generated using the following protocol: a single cell was clamped at a holding potential of −60 mV and Cav current was measured using a stimulus voltage pattern consisting of a 500 ms test pulse from −60 to 60 mV, separated by a 1 s test interval at the holding potential −60 mV. For steady-state channel inactivation, the cell was clamped at a holding potential of −80 mV and stepped to voltage between −60 and 80 mV for 2000 ms to inactivate the Cav current. The cell was then clamped to 10 mV for 250 ms to elicit the Cav current.

### Promoter-binding transcription factor profiling plate array

Promoter-binding transcription factor profiling plate array was performed according to the manufacturer’s instruction. In brief, biotin-labeled transcription factor probes were mixed with nuclear extract with or without the QKI promoter. The transcription factor-DNA complex was separated from free probes using an isolation column, and the bound probes were eluted using elution buffer. Hybridization of eluted probes was performed with hybridization plate. Finally, the bound probe was detected using a streptavidin-HRP conjugate. The QKI promoter sequence used in this assay is shown in the Supplementary materials.

### Western blot

Western blot was performed as previously described [57]. The primary antibodies used were: iNOS (1:1000, Abcam), Arg-1 (1:1000, Abcam), QKI (1:1000, Abcam), CACNA1C (1:200, Santa Cruz), and IL-1β (1:1000, Abcam).

### Statistical analysis

Data are expressed as mean ± SD. An unpaired Student’s *t* test was used for statistical comparison between two groups after the demonstration of homogeneity of variance with an *F* test, and one-way ANOVA was used for comparison of more than two groups. Fisher exact test was used for evaluating the incidence of AF. A *p* value less than 0.05 was considered statistically significant.

## Electronic supplementary material

Below is the link to the electronic supplementary material.

Supplementary material 1 (TIFF 226 kb)

Supplementary material 2 (TIFF 619 kb)

Supplementary material 3 (TIFF 2484 kb)

Supplementary material 4 (DOC 17 kb)
