# Supplementary figures and images for: Cross-talk between macrophages and atrial myocytes in atrial fibrillation
Source: Basic Res Cardiol. 2016 Sep 22;111(6):63. doi: 10.1007/s00395-016-0584-z (PMC5033992; doi:10.1007/s00395-016-0584-z)

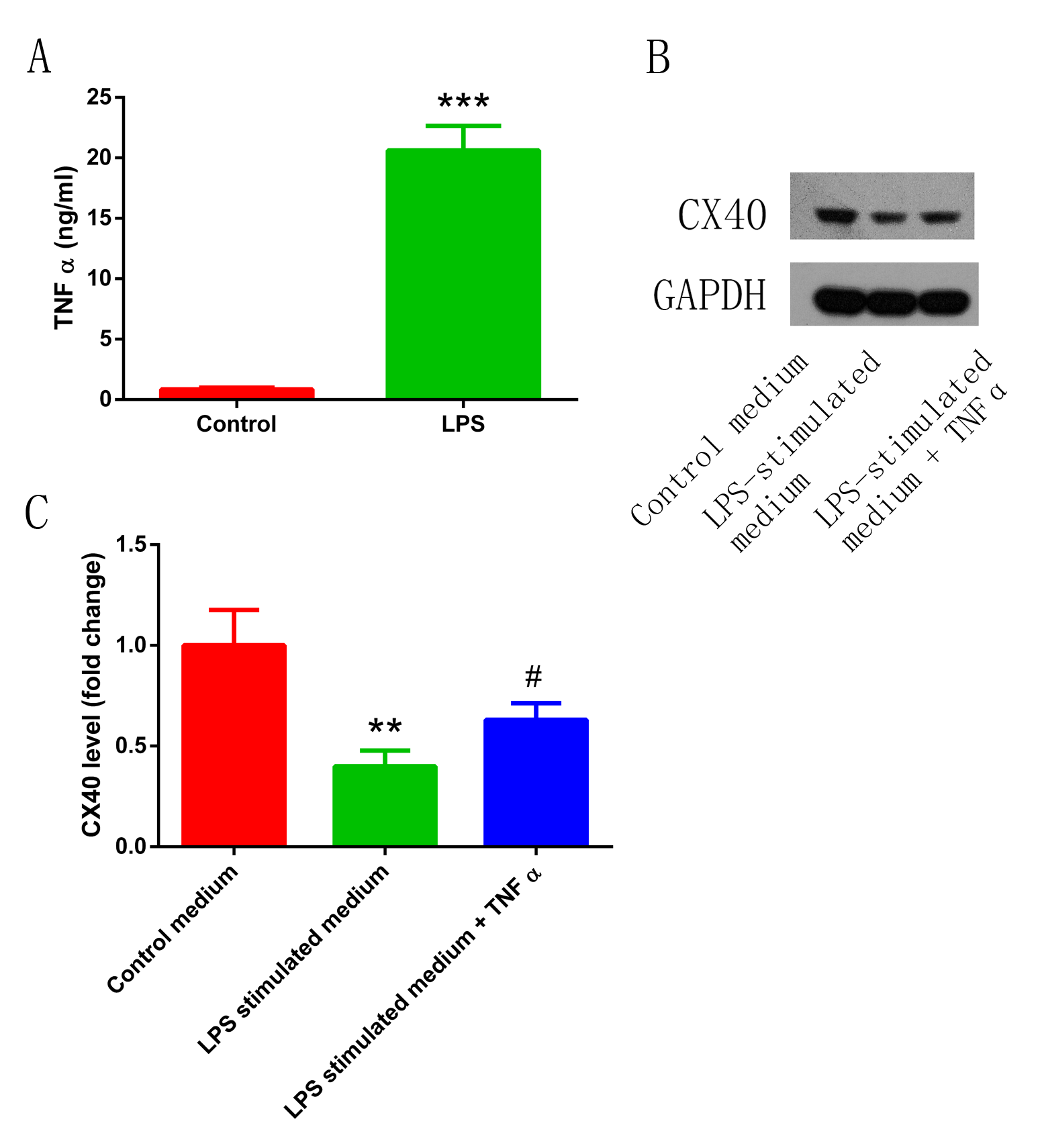

Supplement: Supplementary file 1 — Supplementary material 1 (TIFF 226 kb) [file 395_2016_584_MOESM1_ESM.tif]

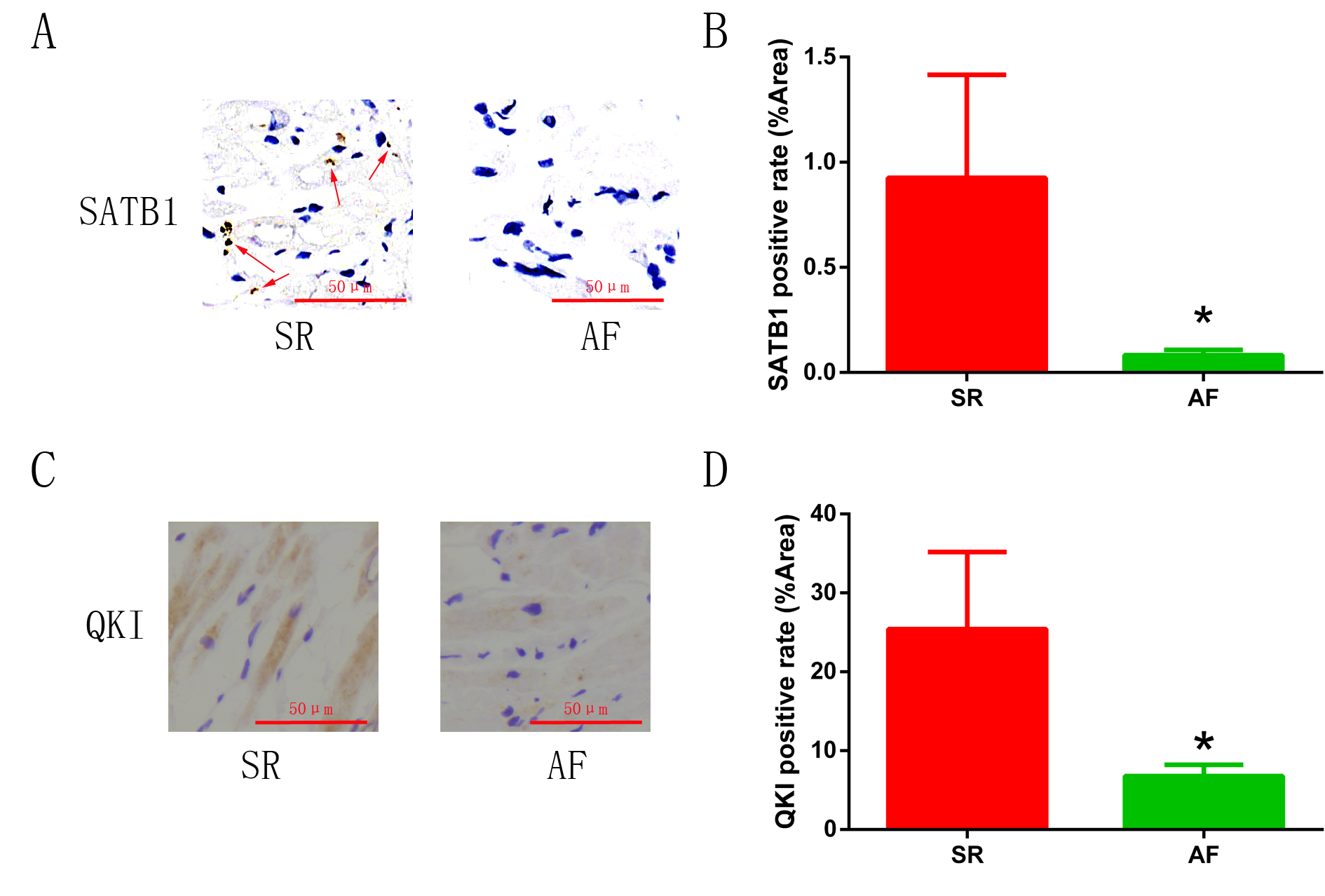

Supplement: Supplementary file 2 — Supplementary material 2 (TIFF 619 kb) [file 395_2016_584_MOESM2_ESM.tif]

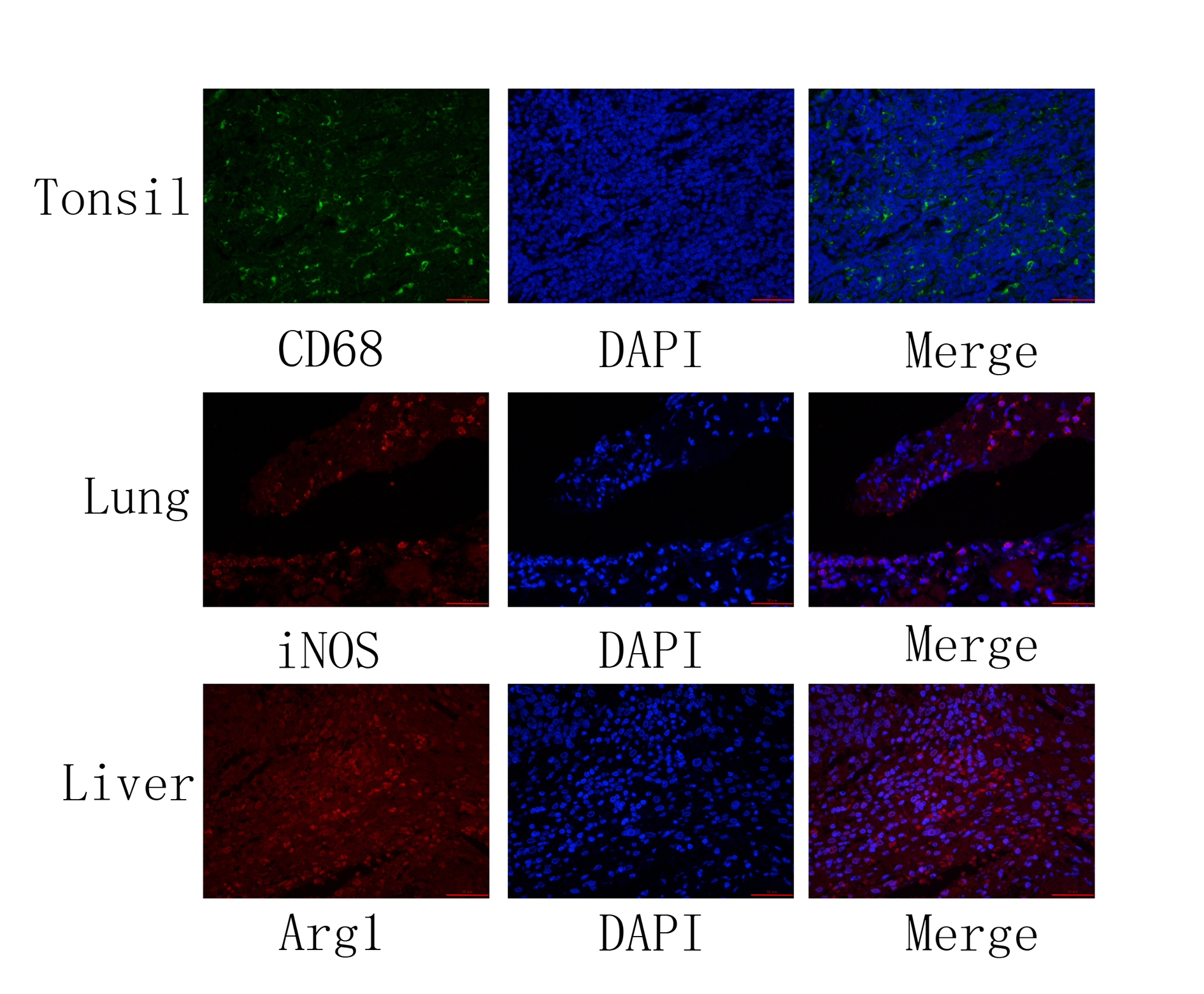

Supplement: Supplementary file 3 — Supplementary material 3 (TIFF 2484 kb) [file 395_2016_584_MOESM3_ESM.tif]
