# Supplementary material for: Cross-talk between macrophages and atrial myocytes in atrial fibrillation
Source: Basic Res Cardiol. 2016 Sep 22;111(6):63. doi: 10.1007/s00395-016-0584-z (PMC5033992; doi:10.1007/s00395-016-0584-z)
Supplement: Supplementary file 4 — Supplementary material 4 (DOC 17 kb) [file 395_2016_584_MOESM4_ESM.doc]

Article title: Cross-talk between macrophages and atrial myocytes in atrial fibrillation

Journal name: Basic research in cardiology

Author names: Zewei Sun, Dongchen Zhou, Xudong Xie, Shuai Wang, Zhen Wang, Wenting Zhao, Hongfei Xu, Liangrong Zheng*

Corresponding author: Liangrong Zheng; Department of Cardiology, The First Affiliated Hospital, College of Medicine, Zhejiang University, No.79 Qingchun Road, Hangzhou 310003, China.

Email: zlrylnn@126.com

Supplementary methods 1:

The QKI promoter sequence used in promoter-binding transcription factor profiling plate array is shown below:

5’-GTCCCGCTCCTCCGTCCGCAGGCCATCTTCGTCCCGCTCCACCCCCACGCAGGCGCGCTCCAGGCCCGCTTCCCCCCCACCCCCCGCCCCCACGGCGCGCTCTCCGCCAGGTGGGGCGGAGCGACCCTCGGAAGGGGCGTGGTCACAGTCCTCGACGCGCGCACGCGCTCTCAGCCTCTCGGCTACGTGTCTGGACACGTGACGCCCACCCGAGAGTCCCCAGTGAAGCAACAGGACGTTTAAAAAAAAAAAATTTTTTTTTTTTTGAGGGGGTAGCGAGGAAGGTGGACGAAGAGGTTTAAGAAAAAAAAAAAAAACCCAAACTATAGTAACGCGCGCGGACGCGAGGCCCGGGCGGCGGCGAGCGCAGCGCGTCGCGGAGAGTCCCGCCGGGATTCCTCTCAGCCGCCGCACCCGACCGCGCCCGCCGCCTCAGCAACAGCGCGCCGACGAGCCAGCGCCGCCGCCCGCGTCCCCCTCAGCCGCGACCCCGGCCCCGCGCGGGCGGCCACCGGCGCGCGTCCTCCCCGCCGCGGCCCGGCCCTGCGCCGGCCGTCCCGCCCGAGCGGCCAGGGGCGGCGGCGCCGCCTGAACTGAGGCGAACTCCGCCGCCAAGTGAGTGAGGAGGAGGAGAGCCGCGGAGGAAGAGGAGGAGCGCAGTCGGAGCGCGGCGGCGGCGGCGGCGGCGGTAACGCGGAGCGCCGCGGCGGACAGCACCGCAGACTCGCGAGCTCCGGCGCAGGCGGCGCGACCGGCCGACGAGCGGCCCCCCGGCTCCTCGCGGCGCAGCGGCCCCGCGCCGCGCTCGGCCCGAGACCCGCCTCTGCCGCCGGCCTCGCGAGGGGAGCCGCGCCGGGCGGCCGAGGAGCGGCGGCCGCGGCGCGTCGGGCCGGGCGAAAGTGCCTGCGGGGGGCGGGCGGGCGCGCGGAGCCGGCCGCCCCGGGGCTCGGCGCGGGAGCCGGAGCGGGAGCCGGCGCGGAGCGGGACGCCGCGTCCCGAGCGGCCGGCGGCCGGAGCTCGCCCCCGCCCCTCCCTCCTCTCCTCCGGCGGCGGCGGCGGCGGGCGGAGTGAGCTGCGGAGCCTGGAAT-3’

**Figure legends**

**Supplementary Figure 1. LPS-stimulated macrophages promoted atrial myocytes electrical remodeling through TNF-α.** (A) TNF-α was increased in the medium of LPS-stimulated macrophages. (B) Co-culture with LPS-stimulated macrophage medium inhibited the CX40 level of atrial myocytes while TNF-αantibody abolished this effect. (C) Statistical result of (B). Data were compiled from three independent experiments. ***p < 0.001, LPS *vs*. control group; **p < 0.01, LPS stimulated medium *vs*. control medium; #p < 0.05, LPS stimulated medium + TNF-α antibody *vs*. LPS stimulated medium

**Supplementary Figure 2. Decreased Satb1 and QKI expression in AF patients.** (A) Decreased Satb1 expression was observed in AF patients. (B) The statistical result of (A). (C) Decreased QKI expression was observed in AF patients. (D) The statistical result of (C). Immunofluorescence was performed on RAA sections obtained from 11 patients with SR and 8 patients with AF. *p < 0.05, AF *vs*. SR.

**Supplementary Figure 3. Positive control staining of CD68, iNOS and Arg1.** Human tonsil, lung and liver served as positive controls of CD68, iNOS and Arg1, respectively. Each antibody worked well in immunofluorescence staining.
